# On the existence of another source of heat production for the earth and planets, and its connection with gravitomagnetism

**DOI:** 10.1186/2193-1801-2-513

**Published:** 2013-10-05

**Authors:** Alexandre Chaloum Elbeze

**Affiliations:** University Pierre et Marie Curie, Campus Jussieu, Pyramide Patio-Students, 24-14-13-23, 4, place Jussieu, 75252 Paris cedex 05, France

**Keywords:** Earth’s surface heat, New source of heat, Reduced heat flow, Earth’s thermal balance, Gravitomagnetism, Radioactive heating, Bulk Silicate Earth (BSE)

## Abstract

Recent revised estimates of the Earth’s surface heat flux are in the order of 47 TW. Given that its internal radiogenic (mantle and crust) heat production is estimated to be around 20 TW, the Earth has a thermal deficit of around 27 TW. This article will try to show that the action of the gravitational field of the Sun on the rotating masses of the Earth is probably the source of another heat production in order of 54TW, which would satisfy the thermal balance of our celestial body and probably explain the reduced heat flow Qo. We reach this conclusion within the framework of gravitation implied by Einstein’s special and general relativity theory (SR, GR). Our results show that it might possible, in principle, to calculate the heat generated by the action of the gravitational field of celestial bodies on the Earth and planets of the Solar System (a phenomenon that is different to that of the gravitational tidal effect from the Sun and the Moon). This result should help physicists to improve and develop new models of the Earth’s heat balance, and suggests that contrary to cooling, the Earth is in a phase of thermal balance, or even reheating.

## Introduction

Approximately fifty per cent of the heat generated by the Earth is thought to be produced by the radioactive decay of elements such as uranium, thorium and their isotopes. Geophysicists estimate heat flow from the Earth’s interior to be in the order of 47 TW (Davies and Davies 2010), which is similar to, but slightly higher than previous estimates (e.g. Pollack et al. 1993; – 44.2 TW ± 1 TW and Jaupart et al. 2007 – 46 TW ± 3 TW ).

What still remains to be understood is the quantity of heat generated from the Earth’s primitive heat and the heat produced through the decay of radioactive elements found in the mantle. The most popular model of radioactive heating is based on the Bulk Silicate Earth (BSE) model (McDonough and Sun 1995), which assumes that radioactive materials, such as uranium and thorium, are found in the Earth’s lithosphere and mantle but not in its iron core. The BSE model also states that the amount of radioactive material can be estimated by studying igneous rocks formed on the Earth and the composition of meteorites.

From this model scientists believe that approximately 20 TW (Mareschal JC et al. (1999)) of heat is created by radioactive decay (Palme and O’Neil 2003 and Bellini et al. 2010), comprised of around 8 TW from uranium (^238^U), 8 TW from thorium (^232^Th) and 4 TW from potassium (^40^ K). Of this, 7 TW is believed to be created in the Earth’s crust and 13 TW in the mantle.

At the same time around 8 TW has been attributed to core dissipation in solid earth. Other heat sources have also been suggested; 39 TW of surface heat flux has been attributed to mantle convection processes, which include approximately 1 TW of latent crystallization heat at the inner core boundary (gravitational energy released by the compression of the core would be of the same order), and residual heat from planetary accretion. Although this initial heat may have rapidly dissipated through the Earth’s superficial layers, slower internal processes would still continue even today (according to some authors this energy has already dissipated).

The heat dissipated by the Earth’s mantle is believed to be around 39 TW, while internal heat production is thought to be up to 21TW. In other words, if the Earth dissipates more heat than it produces, it is cooling. The difference of 18TW (Table 1, Jaupart et al. 2007) can be explained by the secular cooling of the mantle.

**Table 1:** **Mantle energy budget: preferred value and range**

	TW	TW
Oceanic heat loss (300 × 10^6^ km^2^)	32	30−34
Continental heat loss (210 × 10^6^ km^2^)	14	13−15
Total surface heat loss (510 × 10^6^ km^2^)	46	43−49
Radioactive sources (mantle + crust)	20	17−23
Continental heat production (crust + lith. mantle)	7	6−8
Heat flux from convecting mantle	39	35−43
Radioactive heat sources (convecting mantle)	13	9−17
Heat from core	8	5−10^*a*^
Tidal dissipation in solid earth	0.1	
Gravitational energy (differentiation of crust)	0.3	
Total input	21	14−27
Net loss (mantle cooling)	18	8−29
Present cooling rate, K Gy^-1^	118	53−190
Present Urey ratio^*b*^	0.33	0.21−0.49

^*a*^This range includes estimates from core thermodynamics and inference from the perovskite-post-perovskite phase diagram.

^*b*^Urey ratio for the convecting mantle, leaving out crustal heat sources from both heat loss and heat production. The distribution in the range is barely known for most cases and the preferred value is simply the middle one. The cooling rate is computed assuming *Cp* = 1200JK^-1^ kg^-1^.

**Figure 1:**
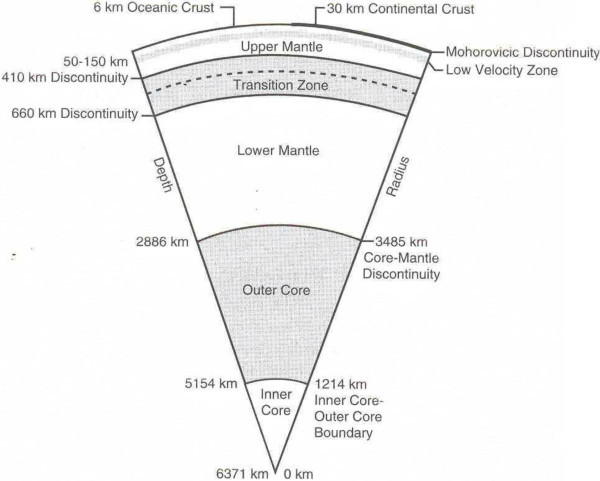
**Simplified plan of the internal structure of the Earth.**

In this paper we argue that there is another potential source of heat that should be taken into account. This heat is created in the Earth’s various layers, mainly the inner and outer core and the mantle (Figure 1) and is the result of the gravitational action of stars (in particular the Sun) and planets on the Earth (and should not be confused with tidal action). We argue that the gravitational influence of the Sun is primordial and far more significant than that of the other planets in the Solar System.

Our argument is based on earlier work (Elbeze 2012) and takes as a starting point results related to the gravitomagnetism framework implied by Einstein’s general relativity theory (GR).

## The lense-thirring effect

According to Einstein’s GR theory (Einstein 1950), the action of the gravitational potential U of a given distribution of mass-energy is described by the coefficients g_μ,ν_., μ,ν. = 0,_1_,2,_3, of the space-time metric tensor. They are determined by solving the fully non-linear field equations of Einstein’s GR theory for the mass-energy content. These equations can be made linear in the weak-field (*U/c*^*2*^ << 1, where *c* is the speed of light in vacuum) and slow-motion (*v/c <<* 1) approximation (see ML Ruggiero and Tartaglia 2002), valid for the Solar System, and appear similar to the linear Maxwell electromagnetism equations and the non-central force *F*_*LT*_, defined as follows:1

Eq. (1) shows the force acting on a test particle of mass *m* induced by the post-Newtonian component ***B***_*g*_ (De Sitter W (1916a)) of the gravitational field in which the particle moves with velocity *v*. ***B***_*g*_ is determined by the mass currents of the matter-energy distribution of the source with mass *M* and comes from the off-diagonal components *g*_*oi*_*, i* =1, 2, 3 of the metric tensor. The gravitational effects induced by mass displacements are collectively named gravitomagnetism. For a central rotating body of mass *M* with angular momentum *S* (parallel to the  axis) radius *r,* and the Newtonian gravitational constant *G* the gravitomagnetic field is given by:2

Eq. (1) shows that the ratio  is proportional to the non central force ***F***_*LT*_. It therefore follows that the gravitomagnetic action of the rotation of the mass *M* is proportional to the ratio . Acceleration *γ*_*LT*_ caused by force *F*_*LT*_ in the Newtonian evaluation can then be formulated as follows, where  is equal to  and  < < 1:3

In the Newtonian context, this residual acceleration  (orthogonal to the direction of angular momentum  and speed ) caused by the Lense–Thirring effect, has a vector component along radius *r* and the of *β* function (projection of the vector  along radius *r*). Which combines with the Newtonian radial acceleration of a test particle of mass *m* along radius *r* to give total acceleration γ as follows:4

If the frame of reference of test mass *m* changes, it is as if *G* (Newton’s gravitational constant) takes the value *G*_*referential*_:5

It therefore follows from Eq. (5) that *G* will vary (Ivashchuk and Melnikov 2002;Melnikov 2007) with *z*(*β*) = 1 + *f* (*β*) as shown here:6

And the relativistic mass *M* varies as a function of *β* as shown here:7

Assuming that *k* is equal to ½ and the term  where *β* is equal to *v/c* and *v/c* < < 1 (here *v* is the projection of the vector  on the radial radius r), replacing mass *M* with its relativist value *M*_*relativistic*_, total relative acceleration is given by:8

Here *v* is the projection of the vector speed  along the radial radius r.

It should be noted that if *G* and *M* are relativistic and depend on speed *ν*, then *r* does not depend on this speed and remains constant in this study.

An interesting characteristic of Eq. (8) is that acceleration is no longer independent of the sign of the velocity *v* of the test particles making up the mass *M* at the source of the gravitational field.

## Complete symmetry for the sun and the planets

**Figure 2:**
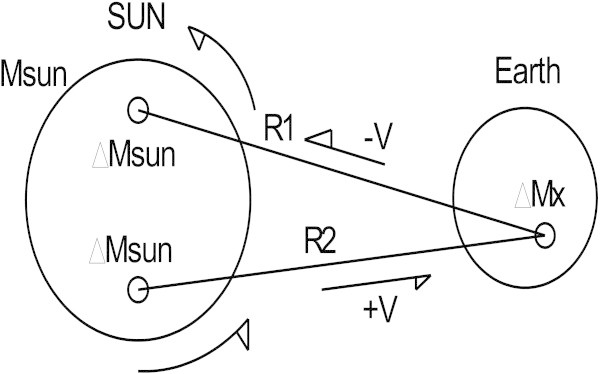
**Cancellation of the Sun’s rotation speed for mass** ***ΔMx.***

We now examine the case of gravitational masses, in particular the Solar System and the Sun whose volumetric expansion and mass are far greater than that of the planets. It is common knowledge that the planets revolve around a stationary Sun which itself rotates upon its axis. Over a short time span, the planets can also be considered as stationary in relation to the Sun and mass *ΔMsun* of the two hemispheres of the Sun moving with speed *+v* or −*v* in relation to the planets (Figure 2).

Speed *ν* is defined as the relative speed between the Sun’s hemispheres and the planet in question, in this case the Earth. This does not take into account the influence of the other planets in the Solar System. The relative speed of the Sun’s rotation seen by a test body *ΔMx* belonging to the Earth is almost zero because it is subject to speeds *+ν* and −*ν* of both hemispheres of the Sun (see Figure 2). Extended to the total mass of the Earth this speed is considered to have no effect on the action of the gravitomagnetic field of the Sun. By applying Eq. (8) and replacing *ν* by zero, acceleration  is equal to Newton’s classic relation.

**Figure 3:**
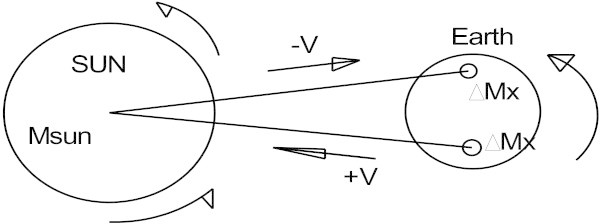
**Relative speed of the Earth’s rotation for mass** ***ΔMx.***

However, this is not completely true as Figure 2 shows the distances *R1* and *R2* are not equal. Nevertheless, for the purposes of our application we will not take this into account. Applying the same reasoning used for the Sun to the Earth, Figure 3 shows the speeds of the Earth’s hemispheres to be +*ν* and −*ν* (the speed of the Earth’s rotation around its axis) and this relative speed is taken into account in the Eq. (8) of the acceleration γ produced by the Sun on the element of mass *ΔMx* of the Earth.

Applying Eq. (8) and replacing *ν* by the speed, along the radius r, of the Earth’s rotation, acceleration γ takes the form:

## Definition of hidden variables

Our calculations are based on a modified value for the radius of the Earth. This modification is described in Elbeze (2012). The Earth’s real radius is defined as *Rrr* and the differential of the real radius as *dRrr*. As explained in Elbeze (op. cit.) the Earth, Sun, planets and the stars in general are complex systems and their apparent dimensions cannot be used directly in calculations.

This is a result of position-dependent hidden variables that maintain these celestial bodies in their respective planetary systems. In the case of the Earth the tilt of its rotation axis with respect to the ecliptic plane defines the real radius *Rrr* used here. Let us assume real radius *Rrr* for the Sun, and the apparent radius *Rcb* (cb for celestial bodies) for the planets of the Solar System, modified data *Ωcb* and experiential data *Ωdata*, which is the projection of the sum of the angles of the axis of rotation and the angle of the orbit on the ecliptic plane.

**Figure 4:**
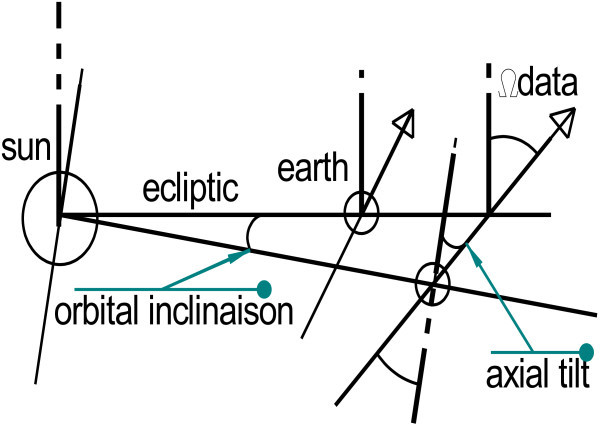
**Definition of the angle** ***Ωcb.***

*Ωcb* and *Ωdata* consist of two data items: the orbital inclination^a^ (Seidelmann et al. 2007), which is the angle (in degrees) between the planet’s orbit around the Sun and the ecliptic plane. The ecliptic plane is defined as the orbital plane of the Earth; therefore the Earth’s inclination is 0. The second factor is the axial tilt^a^ (Seidelmann et al. 2007), which is the angle (in degrees) between the rotational axis of a planet (the imaginary line running through the center of the planet from north to south poles) and its orbital axis around the Sun (see Figure 4).

*Ωcb* (Table 2) shows the corrected angle formed by the planet’s rotational axis and the Earth’s orbital plane (the ecliptic) used in our calculations. Obviously *Ωcb* must be unique for the celestial body in question. A global study of *Ωdata* for orbital inclination and axial tilt led to the use of *Ωcb* as a variable.

**Table 2:** **Modifications of** ***Ωdata*** **for the planets and the Sun (Elbeze** 2012 **)**

i Planets	Angle the ecliptic makes with the projection of the axis of rotation of the planet on the ecliptic ***Ωdata*** (the data observations in radians)	modified ***Ωdata*** as ***Ωcb*** in radians
0 Sun	0.12654	0.00139
1 Mercury	0.12235	0.08221+ π
2 Venus	3.15556	3.12623
3 Earth	0.41015	0.45989
4 Mars	0.87092	0.81071
5 Jupiter	0.07679	0.45789 + π
6 Saturn	0.50964	0.71305
7 Uranus	1.72089	2.54469
8 Neptune	0.52534	0.5435

The real radius (*Rrr*) can be written as:9

And the differential *dRrr* can be written as:10

*Rcb* corresponds to the apparent radius of the celestial body, here the Earth. From Eq. (9) and Eq. (10) it follows that the apparent radius *r* can be replaced by:11

**Figure 5:**
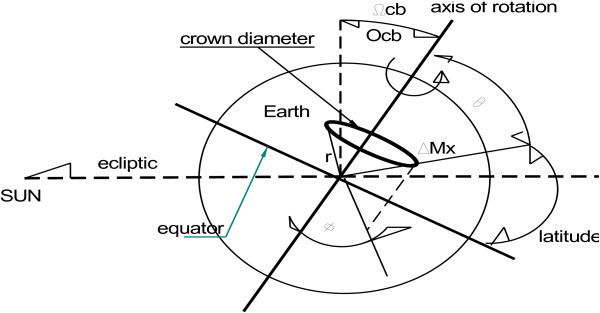
**The Earth’s tilt and the crown diameter.**

This data makes it possible to calculate ***H***_planet_ and ***V***_planet_ (see Figure 5) which are used later in the calculation, as follows:12

**Figure 6:**
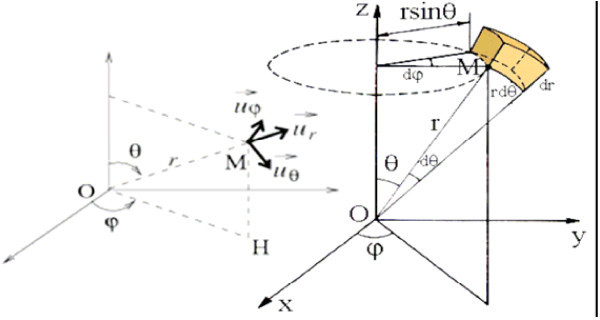
**Polar coordinates of point** ***M*** **of mass** ***ΔMx*** **on the Earth.**

***H***_planet_ is the projection of the crown diameter on the axis which connects the center of the Sun to the center of the planet. ***H***_planet_ is defined by the polar coordinates (Figure 6) *r* and the angles *θ, φ*. ***O****cb* represents the tilt of the axis of the Earth or of the planet on their orbital inclination. In the case of the Earth ***O****cb* is equal to *Ωcb* (see Figure 5).

In the following we calculate heat flow as a function of the latitude of the point in question. So far we have defined an effective radius *Rrr* which is function of *Ωcb* and the constant 62.3. This constant is only valid in the case of a planetary system where the projection of the angle *Ωcb* remains fixed on the ecliptic, as is the case for the Solar System. If we consider this variable angle *Ωcb* to the same planet along the ecliptic, as is the case in heat flow calculations that are a function of latitude, we need to define a function *f* (*θ*) to replace the constant. This fonction *f* (*θ*) is relatively easy to find and takes the following empirical form:13

represents the latitude of the point considered.

**Figure 7:**
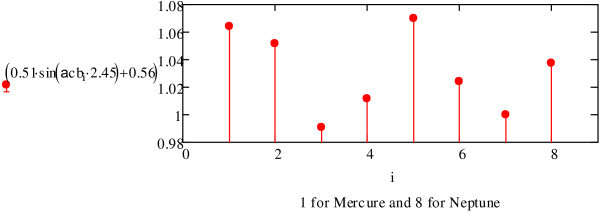
**A calculation of** f ***(*** **θ) where** ***i*** **=1 for Mercury and** ***i*** **= 8 for Neptune.**

It is clear that this function takes an approximate value of 62.3 for all the planets of the Solar System according to their angle *Ωcb* on the ecliptic (see Figure 7). Equations (11) and (12) can be reformulated as:11a12a

In a short time frame (e.g. 24 hours) the speed of the Earth’s rotation is constant, but its direction is variable. Consequently, it is easy to calculate the average relative velocity ***V***_planet_ when a mass *ΔMx* moves from the point farthest from the Sun to the nearest point. It can be represented as:14

***H***_planet_ is the distance traveled in 12 hours, half the time required for a complete revolution of the Earth, from the farthest to the point nearest to the Sun. It should be noted that the real radius *Rrr* is used to calculate ***H***_planet_ and ***V***_planet_. Both ***H***_planet_ and ***V***_planet_ are hidden variables, dependent on the position of the planet in the Solar System and its inclination to the ecliptic (Elbeze 2012).

## Reaction between the sun’s gravitational field and the earth’s rotation

Using the polar coordinates and considering the Earth as having a quasi-continuous density in different parts from the inner core to the upper mantle we will consider mass *ΔMx* whose volume is defined according to the Figure 6.

Obviously, the radius used to calculate the volume and mass of *ΔMx* does not vary according to *Ωcb* as this is a real number and an apparent radius. *dV* is calculated as follows:15

The infinitesimal mass *dm* can be defined as:16

Here *μ* is the density of the zone on the Earth and *r* is the vector radius *r* of the testing mass *ΔMx* (see Figure 6).

Next we calculate the effect of the gravitational field of the Sun on the mass *ΔMx* along its upward or downward trajectory of height ***H***_planet._ From Eq. (8) and using  with *dW* = *dWu* for upward and *dW* = *dWd* for downward for *D1* and *D2* this gives the equations below (16a and 16b).

In order to facilitate the calculation, and not introduce large errors into the final results, the following considers ***H***_planet_ and ***V***_planet_ as acceptable average values. This is preferred to more accurate calculation that takes into account the infinitesimal displacement  and variable speed .

Despite this simplification, which only incurs a slight quantitative error, we must bear in mind the fact that the speed .of the infinitesimal mass dMx has a direction which varies from 0 to 2*π* over 24 hours.

Therefore, the gravitomagnetic force along the radius vector ***H***_planet_ is not nul.16a16b

Equations (16a) and (16b) define the two extreme points of the Earth from the center of the Sun

For *dWu* with speed equal to –*v planet* we have:17

For *dWd* with speed equal to *v planet* we have:18

Where *Dstar* represents the distance from the center of the Sun to the center of the Earth, *Mstar* is the mass of the Sun, *G* is Newton’s constant and *c* is the speed of light. The total gravitational energy of the reaction is given by:19

*Wt* (measured in watts or *J* sec^–1^) represents the total heat created by the gravitational action of the Sun on the planet, here the Earth. The term (24 3600 sec) is the total time taken for a full rotation of the planet.  represents the factor of *dr* as shown earlier (Eq. 11). The calculation can be simplified as follows: 20

In reality, the planets of the Solar System travel along ecliptic orbits and *Dstar* should be replaced by *Dstar* = *a* · (1 − *ex* · cos(*λ*)) or *a* = half major axis = 149, 6 · 10^9^ · *m* For the Earth, *ex* is the eccentricity (0.0167) and λ varies from 0 to 2π (0 in winter and π in summer; the farthest point of the Earth from the Sun).

The distance *R1–R2* (from the center of the planet) represents the depth of the layer used to calculate the energy generated by the reaction with the gravitational field of the star. In the case of the Earth *R1* = 0, *R2* = *Rearth* and  which gives a value of 6.4 · 10^13^ · Watt  for *Wt*.

## Application of *Wt* to the earth

We used data from Figure 1 and internal data densities to calculate the total heat production *Wt* at different depths as shown in Table 3 below:

**Table 3:** **Depths, densities and heat production (** ***Wt*** **) of the Earth’s interior**

Data on the Earth’s Interior from***Wt***					
	Thickness (km)	Density (g/cm^3^)	Wt (10^12^Watt)		±Δ
			λ=0	λ =π	
Crust	30	2.2 to 2.9	0.84	0.69	±0.2
Upper mantle	660	3.8 to 5.4	17.05	15.22	±1.5
Lower mantle	2,226	5.2 to 6.2	32.40	30.40	±2.8
Outer core	2,268	9.9 to 12.2	6.86	6.43	±0.5
Inner core	1,217	12.8 to 13.1	0.04	0.036	
All the Earth	6,371	5.5	59.50	56.00	±5

λ defines the Earth’s position relative to the Sun (see Eq. 27 below).

Although the data used in Table 3 is relatively exact it clearly shows that the dimensions and densities are not completely accurate. If it was the case that the value of 32 TW (18 TW from the upper mantel and 14 TW from the lower mantel) had completely accounted for the 47 TW heat contribution from the gravitational action of the Sun and the heat loss by the Earth, the heat contribution from radioactive elements would be in the order of 15 TW, which is comparable with the value generated by the Bulk Silicate Earth (BSE) model.

All of our calculations of *Wt* only take into account the action of the central star (the Sun) and the position of the Earth with respect to the Sun. However, the fact that the Earth takes an elliptic orbit around the Sun implies that *Wt* varies according to the Earth’s position in space. At the same time, other planets in the Solar System have a gravitational effect on the Earth. This can be easily calculated by generalizing *Wt* and replacing the action of the Sun with that of other planets. The general application of *Wt* and with *νplanet* ≪ *c* can be written as:21

With:2223

Where *cb* is the celestial body subject to the gravitational field of mass *Mstar* (which generates the gravitational field), *Dstar* is the distance between the two bodies in question, *Tcb* is the round trip time of the celestial body, *Hcb* and *vcb* concern the body subject to the gravitational field of the mass *Mstar* calculated using (Eq. 22) and (Eq. 23).

## Relationship between Wt and reduced heat flow

In general we assume that the mantle and crust heat flux is proportional to the average surface heat flux. Pollack and Chapman (1977) argued that mantle heat flux represents 40% of the regional average surface heat flux. Despite the fact that their measures were based on a small dataset, we consider here that they are valid up to a minimum scale of about 300 km (Mareschal and Jaupart 2004). Average heat flow data suggest an empirical relationship of the form:

24where  and  represent average heat flux across the designated area and heat production, *Qo* is reduced heat flow and *b* represents the thickness of a shallow layer enriched by radiogenic elements. Eq. (24) reflects changes in average heat flux on a larger scale (> 200 km) and is based on a very large dataset. It implies that reduced heat flux *Qo* is the same at a certain depth and latitude^b^ of the crust in all areas. The assumed value *Qo* is clearly shown in Eq. (21), which expresses the gravitational action of the Sun on the Earth. This data can be checked against the data provided in Table 4 (above), from the study by Thakur and Blackwell, Huffington Department of Earth Sciences, Southern Methodist University, Dallas, TX.

**Table 4:** **Reduced heat flows for the linear data fit of individual terrain**

N°	Terrain	Reduced Heat Flow mW/m^2^	Latitude	References
1	Baltic Shield	24	66 N	Balling, 1995
2	Brazil Coastal	43.152	25 S	Vitorello et al., 1980
3	Central Australia	48.8	23 S	McLaren et al., 2001
4	Eastern USA Phanerozoic	26.961	40 N	Roy et al., 1968
5	Eastern USA Proterozoic	26.524	41 N	Roy et al., 1968
6	Fennoscandia	23.5	54 N	Kukkonen et al., 2001
7	Maritime	34.54	30 N	Hydman et al., 1979
8	Piedmont	28.61	37 N	Costain et al., 1986
9	Ukraine	24.32	49 N	kutas , 1984
10	Wyoming	26.25	42 N	Decker et al., 1988
11	Yilgarn	33.334	32 S	Jaeger, 1970

In order to compare Eq. (21) with the value of *Qo* (reduced heat flow), we must extend Eq. (21) which calculates heat production due to the gravitational action of the Sun on the Earth, to calculate heat flow up to depths of the order of 500 km, comprising the lithosphere and the upper mantel.

Taking θ and φ to represent the latitude and longitude of the area where average heat flow is measured, this relation can be written as follows, with the latitude measured in degrees:25

The calculation of the gravitational action of the Sun on the Earth is shown in Eq. (21). However, for the calculation of heat flow and heat production, we will base the calculation on a 1 m^2^ column in a lithosphere approximately 550 km deep. In this area, heat transfer mainly occurs through thermal conduction, which enables us to assume that the heat produced as a result of the gravitational effect of the Sun in this area is equal across wide areas and therefore comparable to the reduced heat flow shown in Eq. (24). Heat propagation is lowest in the lower mantle; it is no longer completely the result of thermal conduction but various according to the geography of the area and is comparable to heat production  shown in Eq. (24). We will see later that only a portion of the heat production generated by the gravitational field of the Sun in the lower mantel is taken into account in the calculation of the Earth’s heat loss.

Other explanations for the Earth’s internal heat can be found on the Internet.

We can write:26

Where *EXcb* is the eccentricity of the Earth’s orbit or the planet’s orbit (cb indicating a celestal body), and the angle λ varies from January to August from 0 to π with *Dstar* = 1.496 · 10^11^*m* = *a*, where *a* is equal to half major axis of the eliptic orbit of the planet, and:27

Δ*R* is a basic unit of distance. It can be a meter or take an arbitrary value. Here, we use the meter because it is directly related to the unit area *m*^*2*^ or the unit volume *m*^*3*^ which leads to a definition of heat flow and heat production in (*mW*/*m*^2^ or *μW*/*m*^3^.).

Finally, heat production  of the gravitational action of the Sun on the Earth can be written as follows:28

For example, according to Eq. (28) at 23° north, heat production from the gravitational action of the Sun on the Earth can be written as:29

**Figure 8:**
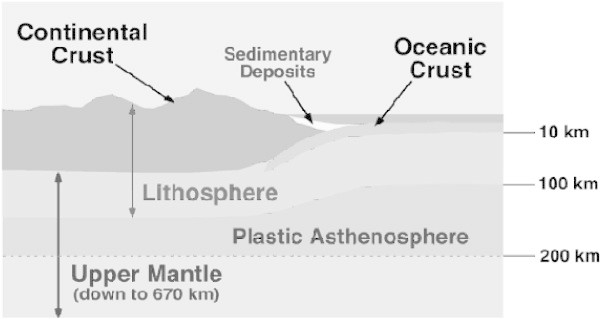
**Structure of the Earth's crust and top most layer of the upper mantle.**

In Eq. (29),  enables heat production and heat flow in a volume of 1 m^3^ to be calculated. Consequently, for a lithosphere of thickness 558 km (Pinet C et al. (1991)) at 23° north the calculation is as follows:

This value of 558 km is close to the 660 km depth of the Earth’s lithosphere and upper mantel (see Figure 8) and λ = 0 (see Eq. 26).

**Figure 9:**
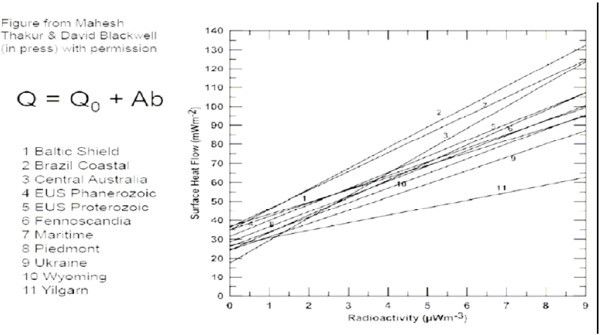
**The linear fit line for individual Q-A data for different terrain of the world.**

**Figure 10:**
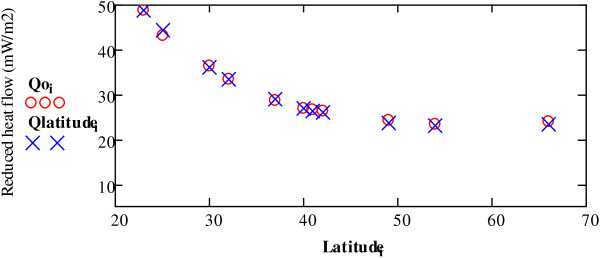
**Application of** ***Q(latitude)*** **in regards to the data** ***Qoi.***

If ***Q****(latitude)* is applied to the data in Table 4, and Figure 9, we can check whether *Q(latitude*) does in fact represent the reduced heat flow *Qo* found in Eq. (24) for regions at different latitudes. In fact Figure 10 shows that the gravitational action of the surrounding planets and the Sun heat the Earth in the same proportions as the reduced heat flow *Qo*.

Earlier literature on the Earth’s heat sources has also suggested the possibility of an external heat source. For example, Jaupart et al. (2007) comment that *Qo* could be due to an external input of heat and differences of the radiogenic heat of the Earth’s crust.

**Figure 11:**
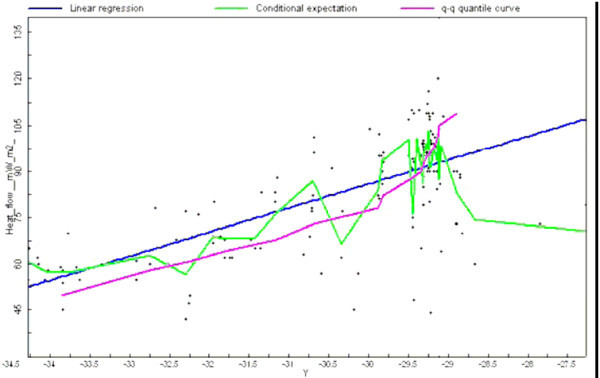
**A heat flow values (y axis) and latitude (x axis).**

It is interesting to note that Figure 10 shows that reduced heat flow decreases at higher latitudes. This is not unusual; several other authors have noted this phenomenon. For example, Figure 11 shows results from a study of the north-northwest of Western Australia (Perth Basin) carried out by the company Hot Dry Rocks Pty Ltd (2008).

## Reaction between the planets’ gravitational field and the earth’s rotation

From Eq. (21), we can calculate the gravitational effect of the planets and the Moon on the Earth. To do this we replace the data relative to the effect of the Sun on the Earth with those of the planet or satellite in question (e.g. the Moon). According to Table 2 the values to be used are *i* = 1 for Mercury, *i* = 8 for Neptune and *i* = 3 for the Moon.

**Figure 12:**
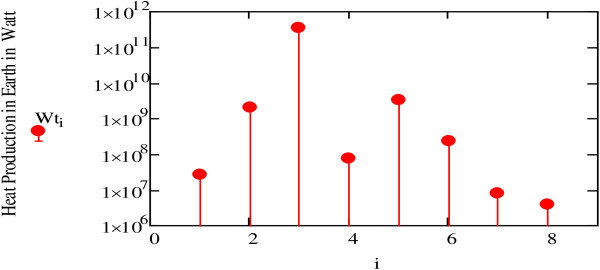
**Calculation of the gravitational action of planets on the Earth (** ***Wt).***

The graph shown in Figure 12 takes into account the minimum distance between the planet and the Earth (i.e. the distance from the Sun to the planet -the distance from the Earth to the Sun).

These calculations assume that the distances between the planets and the Earth remain constant over the period of the Earth’s rotation. Although we know that this is not the case, the values of *Wt* shown in Figure 12 provide a relatively precise glimpse of the heat generated in the Earth. The effect of the Moon is the strongest, generating about 1 TW (particularly compared to tidal dissipation in solid earth of about 0.1 TW).

**Figure 13:**
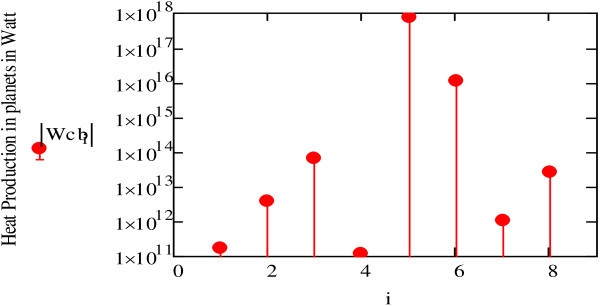
**Calculation of** ***Wt*** **the gravitational action of the Sun on the planets.**

As before, we can calculate the gravitational action of the Sun on the planets in the Solar System. To do this, we apply Eq. (21), taking into account eccentric planetary orbits, to the relationship described in Eq. (26). The results are shown in the graph (Figure 13).

**Figure 14:**
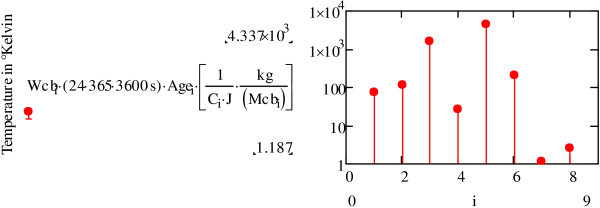
**Calculation of the gravitational action of the Sun on the planets.**

We can then calculate the temperature brought about by the gravitational action of the Sun on the planets of the Solar System for planets aged about 5 · 10^9^ years. Using Eq. (21) we obtain the graph (Figure 14).

*C*_*i*_ is heat capacity measured in *Jkg*^− 1^ · *K*^− 1^; *Mcb* is the mass of the planet or celestial body. The data shown in this graph is imprecise as the value of *C*_*i*_ is not well established.

## Conclusion

Extending earlier studies on the rotation of planets (Elbeze 2012) and particularly the relativistic effect of gravitational action (see Eq. 8 and following) this paper shows that there is another heat source, external to the Earth itself and the action of its radioactive elements. Our calculations suggest that the gravitational effect of the Sun on the Earth generates a total power equal to about 54 TW.

This external heat is due to the action of land masses moving in the gravitational field of the Sun, and depends on the relative speed ±*v* (velocity depends on the rotation of the Earth on its axis). This occurs because there is an asymmetry between the direction of the relative speed and its effect on the moving masses (as described by Eq. 8 and following). Infact it is an example of the gravitomagnetism phenomenon described in the study by Elbeze (2012).

This study has shown that the production of heat in the lithosphere exactly matched the reduced heat flow *Qo* shown in Figure 10, and the heat lost from the lower mantle forms part of the overall heat lost by the Earth. However, if the heat flow created in the lower mantel does form part of the total 47 TW of heat lost by the Earth, then heat produced by radioactive substances in the upper mantle must be less than the current estimate of 20 TW. Similarly, if the gravitational action of the Sun in the lower mantel creates a heat loss by the Earth of about 14 TW, the production of radiogenic heat would be about 15 TW (or less), which is comparable with estimates based on the Bulk Silicate Earth value (BSE) model. The 14 TW produced by the gravitational action of the Sun on the Earth would vary from one area to another depending on the distribution of sedimentary rocks. This energy would add to the part of the heat produced by radioactivity found in the crust and the lower and upper mantels to form the heat flow lost by the Earth. In this case, the balance of the Earth’s heat production would be positive, rather than negative. The overall effect of the gravitational action of the Sun on the Earth would be to increase heat generation by about 25 TW (see Table 3), which corresponds to an increase in temperature of the order of 170° K per billion years.

## Endnotes

^a^Planetary fact sheet; can be found on the Internet.

^b^Latitude is a geographic coordinate that specifies the north–south position of a point on the Earth’s surface. Lines of constant latitude (parallels) run east–west parallel to the Equator. Latitude is an angle which ranges from 0° at the Equator to 90° at the north and south poles.
